# Case report: Ectopic corpus cavernosum presented as bladder tumor in a 3-year-old boy

**DOI:** 10.3389/fonc.2024.1308493

**Published:** 2024-02-12

**Authors:** Jia-gui Chai, Yan-liang Zhao, Si-fan Yin, Zhi-yuan Yin, Shen-zhao Zhao, Run-lin Feng, Chang-xing Ke

**Affiliations:** ^1^ Department of Urology, The Second Affiliated Hospital of Kunming Medical University, Kunming, China; ^2^ Department of Urology, People’s Hospital of Xiangyun County, Dali, China; ^3^ Department of Pathology, The Second Affiliated Hospital of Kunming Medical University, Kunming, China

**Keywords:** ectopic corpus cavernosum, bladder tumor, treatment, pathology, children

## Abstract

**Background:**

Ectopic tissue is rarely found in the bladder for adults. Currently, there have been reports of ectopic prostate and colon tissue in the bladder. These ectopic tissues are manifested as a bladder mass and cause lower urinary tract symptoms. However, the ectopic corpus cavernosum in the bladder has never been reported, and its clinical characteristics and treatment have not been explored yet.

**Case summary:**

A 3-year-old boy was admitted to the hospital due to 1 month of urinary frequency. The physical examination was unremarkable. Urine analysis from other hospitals showed an elevated urine white blood cell count of 17.9/ul. In addition, ultrasound indicated a possible bladder mass. CT and MRI showed a well-margined lesion (1.9×1.9 cm) in the bladder trigone. Through preoperative imaging, we diagnosed a bladder tumor (inclined towards benign). The transurethral resection of the bladder tumor was performed. Unfortunately, the surgery was unsuccessful due to the difficulty in removing the excised tissue through the urethra. Subsequently, bladder incision and tumor resection were performed. The tumor was successfully removed. Surprisingly, the postoperative pathology showed that the tumor tissue was corpus cavernosum. The pathological diagnosis was ectopic corpus cavernosum in the bladder. No complications were found after the operation, and no recurrence was observed during follow-up.

**Conclusion:**

The ectopic corpus cavernosum in the bladder has never been reported for children, which is presented as a benign tumor with rapid proliferation and large size. Surgery is recommended. However, the transurethral resection of bladder tumors is difficult to perform due to narrow urethra and limited surgical instruments. Bladder incision and tumor resection may be preferred.

## Introduction

Ectopic tissue is rarely found in the bladder (1–3). Currently, there have been reports of ectopic prostate and colon tissue in the bladder (1, 2). However, to my knowledge, the ectopic corpus cavernosum in the bladder has never been described before. More importantly, when symptoms occur, problems may arise from the therapy. In this article, we present the ectopic corpus cavernosum manifested as a bladder tumor and share its clinical features and treatment strategy.

## Case introduction

**Figure 1:**
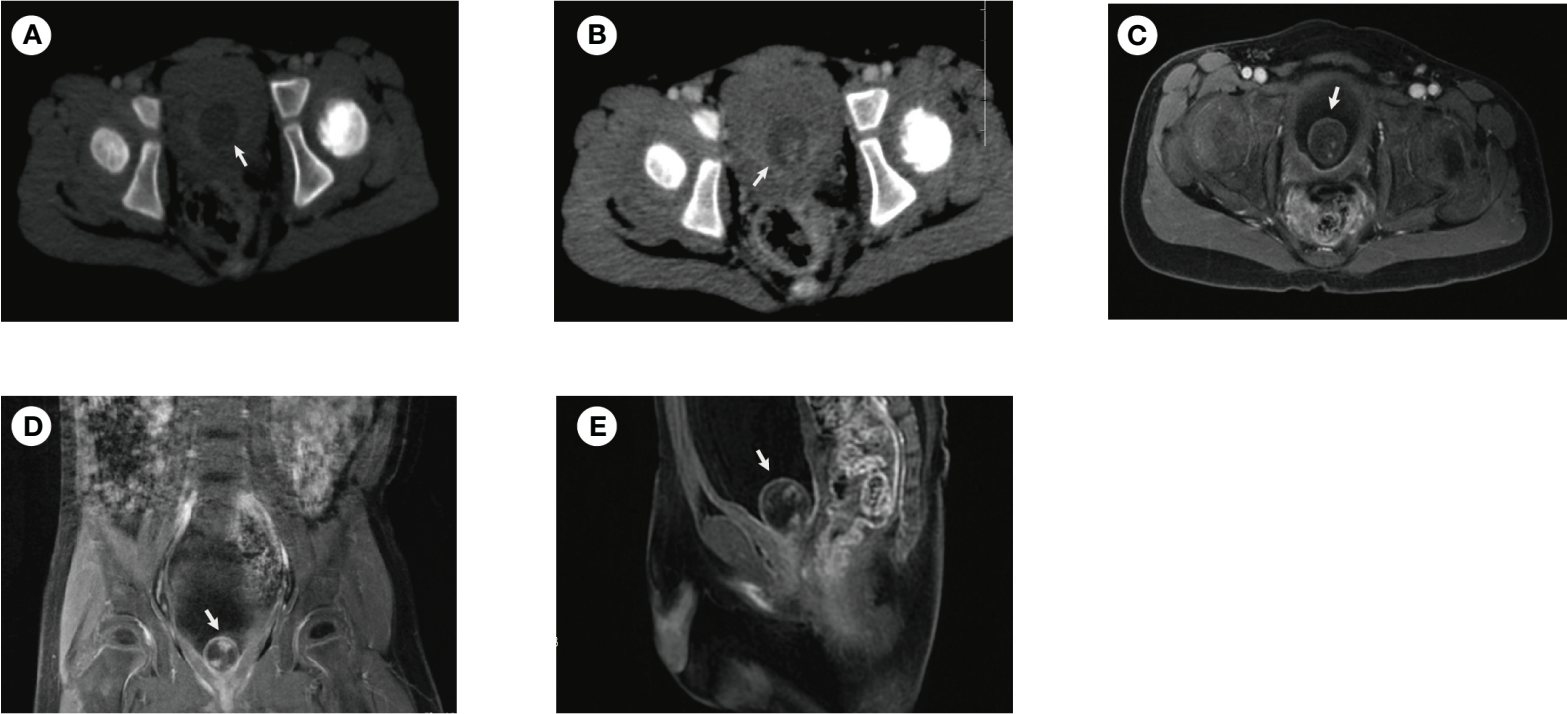
CT and MRI of the patient before surgery. **(A)** CT plain scan revealed a round, low-density, well-margined bladder lesion (1.9×1.8cm). **(B)** CT enhanced scan showed moderate round enhancement of the lesion capsule and small flake-enhanced high-density shadows in the lesion parenchyma. MRI showed a cystic lesion (1.9×1.9 cm) with an annular low-signal of the lesion capsule and a small flake-enhanced lesion parenchyma in the transverse **(C)**, coronal **(D)**, and sagittal planes **(E)**. The white arrow in the image indicates the lesion.

A 3-year-old boy was admitted to the hospital due to 1 month of urinary frequency. The physical examination was unremarkable. Urine analysis from other hospitals showed an elevated urine white blood cell count of 17.9/ul. In addition, ultrasound indicated a possible bladder mass. The patient visited our hospital and further examination was cautiously recommended. CT plain scan revealed a round, low-density, well-margined bladder lesion (1.9×1.8 cm) (**Figure 1A**). CT enhanced scan showed a moderate round enhancement of the lesion capsule and small flake-enhanced high-density shadows in the lesion parenchyma (**Figure 1B**). However, CT could not clearly show the lesion and its surroundings, so MRI with high resolution for soft tissue was recommended. MRI scan showed a cystic lesion (1.9×1.9 cm) with an annular low-signal of the lesion capsule and a small-flake enhanced lesion parenchyma at bladder trigone (**Figures 1C–E**).

We combined with preoperative imaging and diagnosed a bladder tumor (inclined towards benign). We took into account that the tumor was large and the patient was symptomatic. Surgery was recommended.

The transurethral resection of bladder tumor was performed. We initially used a Wolf 4.5F/6.5F rigid ureteroscope (Germany) to enter the bladder and observed a smooth surface, pedicled, single tumor of about 2 cm located in the bladder trigone. Subsequently, we attempted transurethral resection of the tumor. Specifically, thulium lasers (Cyber Tm 2μm thulium laser surgical system, Italy) were used to cut tumor. Bleeding was minimal due to real-time hemostasis at the time of cutting. A Wolf 5F lithotomy forceps (Germany) was used to remove the lump. Unfortunately, transurethral removal of the lump was difficult due to its large size. Subsequently, bladder incision and tumor resection were performed. Specifically, a bladder puncture was performed 1.5 cm above the pubic bone by 18G puncture needle (Bard, USA), and an access was established by 20F Percutaneous Access Set (Bard, USA). This process is similar to the access establishment of percutaneous nephrolithotomy. A Wolf 4.5F/6.5F rigid ureteroscope (Germany) was inserted along the access into the bladder. The thulium laser (Cyber Tm 2μm thulium laser surgical system, Italy) was again used to cut the lump into small pieces and a Wolf 5F lithotomy forceps (Germany) was used to remove the small pieces. The tumor was successfully removed. The total duration of the surgery was 2.8 hours. The F10 Foley catheter was retained for 14 days. When the incision had healed well and there were no symptoms such as hematuria, the catheter was removed. No complications were found after the operation.

**Figure 2:**
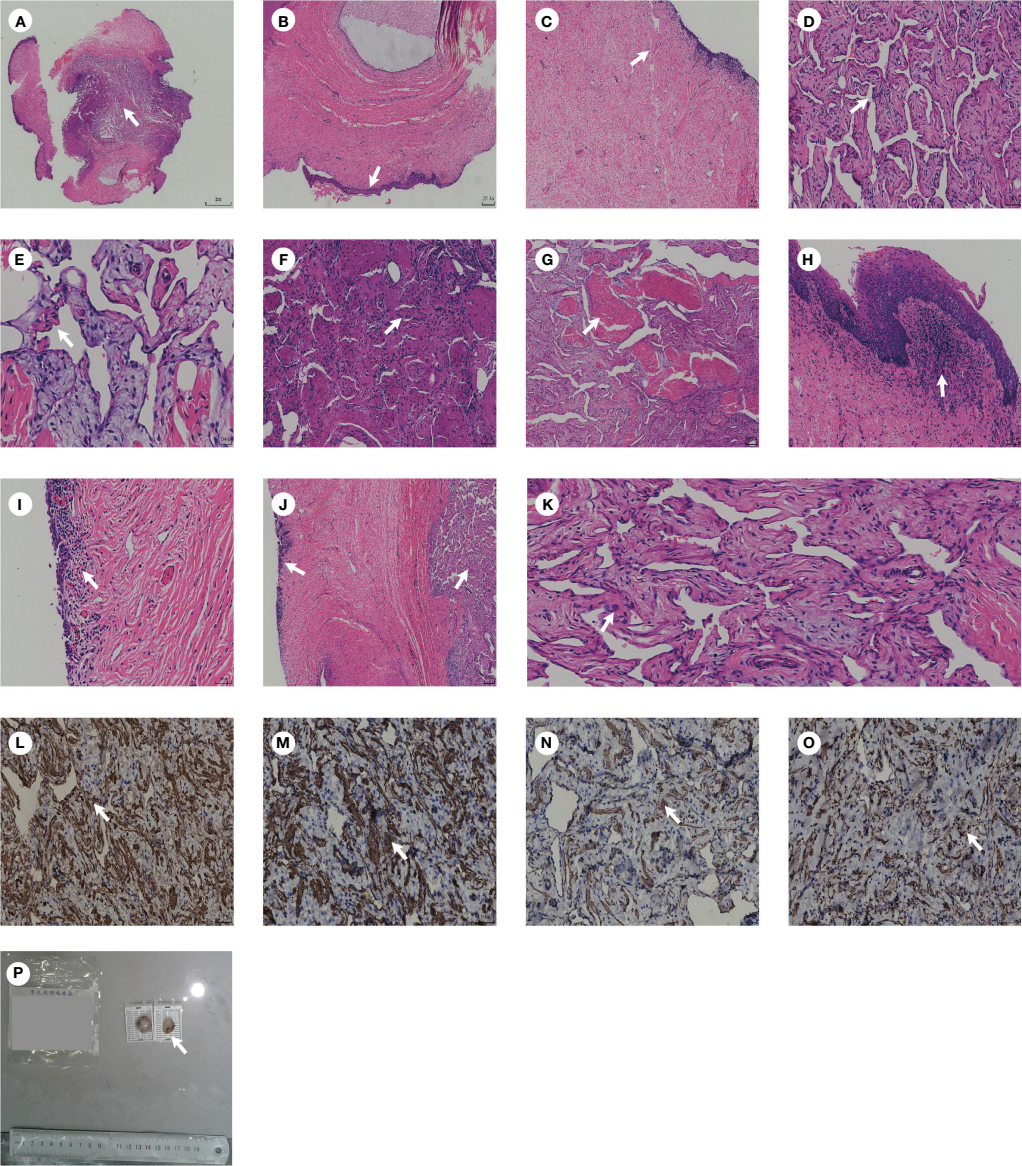
** **A diagram of the pathology. **(A)** Pathological morphology of tissue under low magnification. **(B)** Multilayered squamous epithelium can be seen within the tissue. **(C)** Fibrous connective tissue can be seen within the tissue. **(D, E)** Vascular network lacunae can be seen within the tissue. **(F)** Nerve tissue can be seen within the tissue. **(G)** Smooth muscle can be seen within the tissue. **(H)** Chronic inflammatory cells can be seen within the tissue. **(I)** Bladder mucosa can be seen within the tissue. **(J)** Bladder mucosa and corpus cavernosum can be seen within the tissue. **(K)** Smooth muscle under high magnification can be seen within the tissue. **(L)** Calponin (+). **(M)** actin (+). **(N)** SMA (+). **(O)** Desmin (+). **(P)** A gross specimen of bladder mass. The white arrows in the image indicate typical pathological sections.

**Figure 3:**
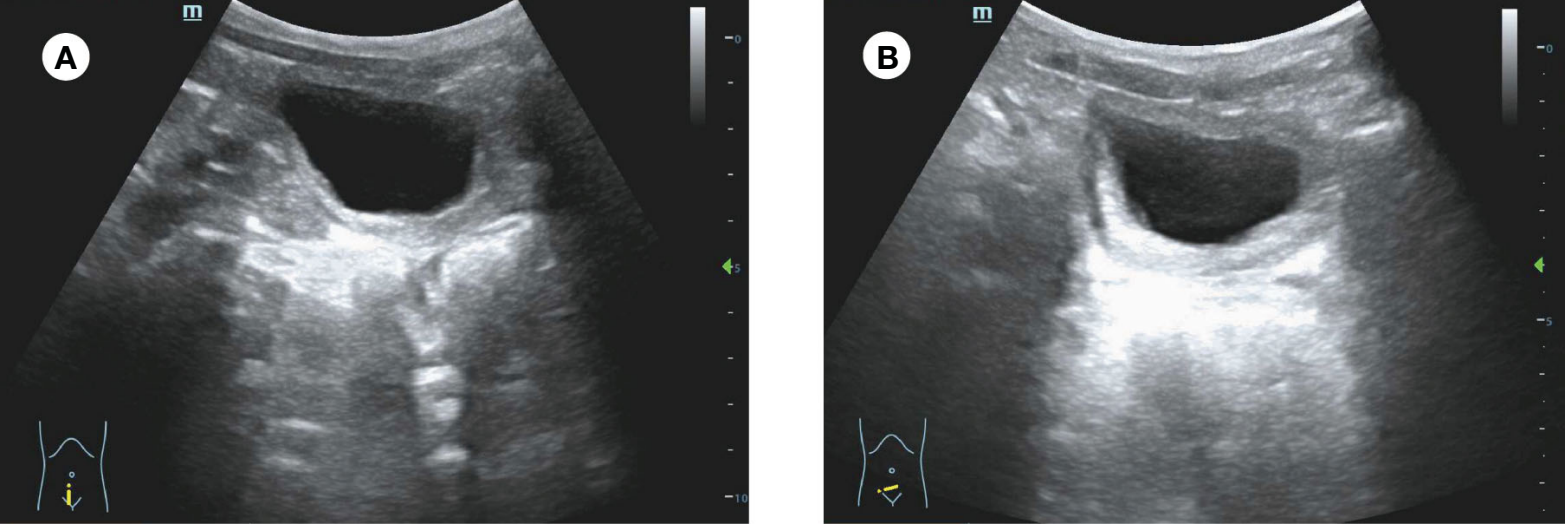
Ultrasound of the patient 10 months after surgery. **(A, B)** No significant abnormalities were found in the bladder by ultrasound.

Interestingly, postoperative pathology showed that the surface of the tissue was covered with multilayered squamous epithelium, with fibrous connective tissue and vascular network lacunae found in large amounts in the parenchyma. In addition, chronic inflammatory cells, nerve tissue, and smooth muscle bundles can be seen (**Figure 2**). This suggests that the tissue is the corpus cavernosum. Immunohistochemistry showed CD31 (+), CD34 (+), CK pan (+), F-VIII (+), Ki-67 (<3%), VIM (+), D2-40 (-), P53 (-), ERG (+), CK20 (-), CK5/6 (-), CK7 (+), GATA3 (-), Calponin (+), actin (+), SMA (+) and Desmin (+). We considered the final diagnosis to be ectopic corpus cavernosum in the bladder. The patient was discharged 5 days after surgery. Ultrasound showed no significant abnormalities in the bladder 10 months after surgery (**Figure 3**) and the patient had no symptoms of urinary frequency. At present, the patient is still being followed up.

**Figure 4:**
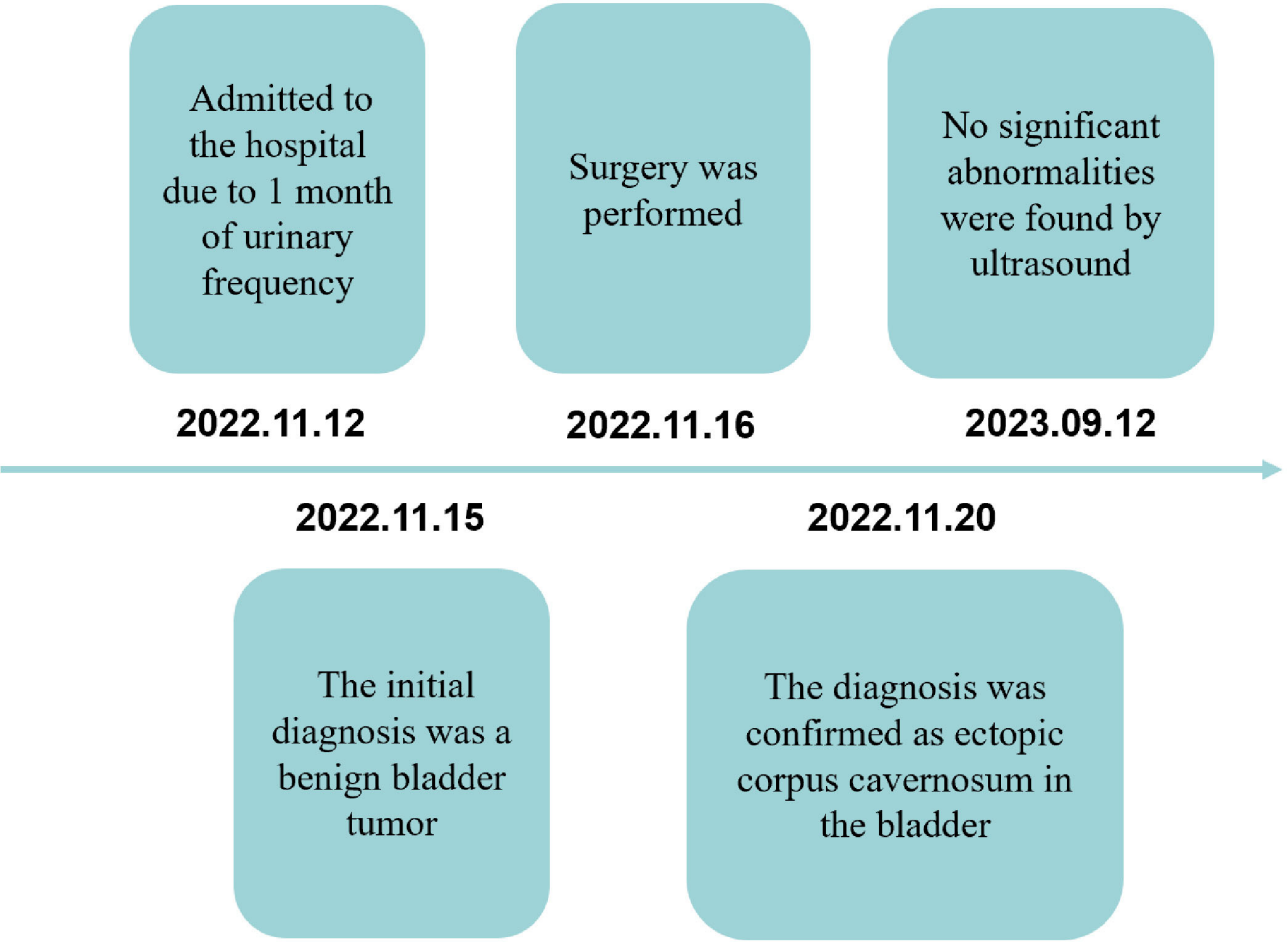
Timeline.

Timeline (**Figure 4**): A 3-year-old boy was admitted to the hospital due to 1 month of urinary frequency—The initial diagnosis was a benign bladder tumor on the 3rd hospitalization day—Surgery was performed on the 4th hospitalization day—The diagnosis was confirmed as ectopic corpus cavernosum in the bladder on the 4th day after operation—No significant abnormalities were found during follow-up.

## Related literature learning

Ectopic tissue is rarely found in the bladder, but there are still some reports, including ectopic prostate and colon tissue (1–3). Although they reported that ectopic tissue formed benign lesions in the bladder, the origin theory of ectopic tissue remains controversial. Currently, there are several theoretical explanations, including the migration and misplacement of normal tissue, the persistence of embryonic remnants, and metaplastic change caused by chronic inflammation. For the ectopic prostate in the bladder, Kim et al. (3) found that it may be related to the migration of prostate tissue and the persistence of embryonic remnants due to pathological and immunohistochemical similarities in normal and ectopic prostate tissues. However, Hasegawa et al. (1) found that colon ectopia is related to bladder cell metaplasia due to pathological support for cell metaplasia and chronic inflammatory infiltration.

In the present case, no metaplasia was observed despite pathological evidence of chronic inflammatory cell infiltration (**Figure 2H**). In addition, immunohistochemical and histological similarities are observed in normal corpus cavernosum and ectopic tissues. However, from the perspective of embryonic development, the migration of the corpus cavernosum to the bladder trigone is difficult to explain. We speculate that it may be a persistent congenital presence of the corpus cavernosum in the bladder trigone, but the cause of this congenital presence is unclear.

The clinical manifestation of ectopic corpus cavernosum in the bladder is urinary frequency. However, this is non-specific due to most pediatric bladder tumors causing urinary frequency or hematuria (2, 4, 5). Imaging is needed to confirm the diagnosis (6, 7). Compared to adults, the first-line imaging tool for assessing pediatric bladder lesions is ultrasonography, which is followed by a cross-sectional imaging examination such as computed tomography or magnetic resonance imaging if the origin of the mass is unclear or if distant spread is suspected (8, 9). It should be noted that MRI is superior to CT because of radiation in pediatric population (it is non-normative for this patient to have both CT and MRI performed). In the present case, Ultrasound indicated a possible bladder mass. MRI showed a cystic lesion with an annular low-signal of the lesion capsule and a small flake-enhanced lesion parenchyma at bladder trigone, which suggests bladder tumor (inclined towards benign).

Furthermore, although biopsy is important to determining diagnosis, a preoperative biopsy may not be necessary due to the inability of children to cooperate in completing the biopsy under non-general anesthesia and the rarity of malignant transformation of benign bladder tumors in children (2, 10–12). We use a Wolf 4.5F/6.5F rigid ureteroscope (Germany) for sampling during surgery, rather than preoperative biopsy, which may be appropriate for this patient (8, 9). In addition, we attempted to excise the tumor as extensively as possible during surgery, while performing postoperative biopsy (the tumor base was not individually biopsied due to consideration of benign mass). However, strictly speaking, for bladder tumors with unclear nature, intraoperative frozen and/or individual base biopsy are necessary. In this patient, it is non-normative to not perform intraoperative frozen or individual tumor base biopsy (although preoperative imaging and intraoperative endoscopy suggest that it may be a benign tumor) (9). In addition, urine cytology is important to help diagnose bladder tumors (9). But this patient was not performed because the imaging indicated that the lesion might be benign.

Pediatric bladder tumors can be divided into those that originate from the bladder epithelium, known as urothelial neoplasms, and mesenchymal bladder neoplasms, which are more prevalent. The common bladder malignancy in children such as rhabdomyosarcoma, papillary urothelial neoplasm of low malignant potential needs to be differentiated. In addition, some uncommon tumors, such as transitional cell carcinoma of the bladder, inflammatory myofibroblastic tumor of the urinary bladder, also need to be differentiated (8, 9). In addition, although the lesion was identified as ectopic corpus cavernosum in the bladder (we considered it a benign mass), it cannot be specifically classified as a specific type of bladder tumor due to the lack of observation of the long-term biological behavior of the lesion. Nonetheless, this lesion still needs to be differentiated from other bladder tumors.

The ectopic tissue in the bladder is presented as a benign mass, which is rarely reported to undergo malignant transformation (2, 11, 12). Therefore, most scholars support that when asymptomatic, no additional treatment is needed (2, 11, 12). However, when the ectopic tissue in the bladder shows symptoms such as hematuria, frequent urination, or imaging suggesting malignancy, surgical treatment is recommended (3). It is reported that the transurethral resection of bladder masses achieved good surgical results (3). However, in this patient, we attempted transurethral resection of bladder masses but failed due to narrow urethra and limited surgical instruments, which prevented the removal of the excised tissue. We subsequently chose bladder incision and tumor resection, which proved successful. For tumors of a similar size in children, bladder incision and tumor resection may be preferred.

Interestingly, postoperative pathology showed that the typical histological presentation of the bladder lesion was the same as that of corpus cavernosum, indicating the ectopic corpus cavernosum in the bladder, which had never been reported before. The immunohistochemistry was characterized by CD31 (+), CD34 (+), and VIM (+), which suggested angiogenesis within the mass. However, Ki-67 (< 3%) and histology showed no atypia, indicating that the mass was more likely to be benign (13). Therefore, we speculate that the ectopic corpus cavernosum in the bladder may be a benign tumor with rapid proliferation.

In conclusion, the ectopic corpus cavernosum in the bladder has never been reported for children, which is presented as a benign tumor with rapid proliferation and large size. Surgery is recommended. However, the transurethral resection of bladder tumors is difficult to perform due to narrow urethra and limited surgical instruments. Bladder incision and tumor resection may be preferred.

## Data availability statement

The raw data supporting the conclusions of this article will be made available by the authors, without undue reservation.

## Ethics statement

The studies involving humans were approved by Ethics Committee of the Second Affiliated Hospital of Kunming Medical University. The studies were conducted in accordance with the local legislation and institutional requirements. Written informed consent for participation in this study was provided by the participants’ legal guardians/next of kin. Written informed consent was obtained from the individual(s), and minor(s)’ legal guardian/next of kin, for the publication of any potentially identifiable images or data included in this article.

## Author contributions

J-GC: Writing – original draft. Y-LZ: Writing – original draft. S-FY: Writing – original draft. Z-YY: Writing – review & editing. S-ZZ: Writing – review & editing. R-LF: Writing – review & editing. C-XK: Writing – review & editing.
